# Duplicate publication of articles used in meta-analysis in Korea

**DOI:** 10.1186/2193-1801-3-182

**Published:** 2014-04-09

**Authors:** Whan-Seok Choi, Sang-Wook Song, Sun-Myeong Ock, Chul-Min Kim, JungBok Lee, Woo-Jin Chang, Se-Hong Kim

**Affiliations:** Department of family Medicine, College of Medicine, The Catholic University of Korea, 505 Banpo-Dong, Seocho-Gu, Seoul, 137-701 Korea; Department of Clinical Epidemiology & Biostatistics, Asan Medical Center, University of Ulsan College of Medicine, Seoul, Korea; Medical Library, The Catholic University of Korea, Seoul, Korea; Department of Radiology, Keck School of Medicine, University of Southern California, Los Angeles, USA

**Keywords:** Duplicate publication, Meta-analysis, Korea, Bias

## Abstract

With the increasing use of meta-analysis, duplicate publication of original research is particularly problematic. Duplicate publication can result in an inappropriate weighting of the study results. The purpose of our study was to assess the incidence and characteristics of duplicate publications in Korea, and to estimate the impact of duplicate publication on meta-analyses. The meta-analysis literature written by Korean authors was searched using the online search engines PubMed, KMbase, and KoreaMed. Duplication patterns were classified into the following 4 combinations: identical samples and identical outcomes (copy), identical samples and different outcomes (fragmentation), increased samples and identical outcomes (imalas), and decreased samples and identical outcomes (disaggregation). To estimate the multiple publication bias, we performed a meta-analysis with and without duplicated data. We estimated that 6 (6.9%) of the 86 analyzed meta-analyses included duplicate publications, and 6 of the 1,194 articles (0.5%) used in the meta-analyses were duplicate publications. In this study, duplicate publications were usually due to disaggregation and overlapping (imalas) publications. Of 6 duplicated articles, 1 was considered a copy (16.6%); 1, a fragmentation (16.6%); 2, imalas (33.3%); and 2, disaggregations (33.3%). There was an increase in the mean effect size and fail-safe number with duplicated data. Our study found only 6 instances of duplicate publication after analyzing 1,194 articles used in meta-analyses written by Korean authors. However, 6.9% of the meta-analyses included duplicate publications. Our findings suggest that meta-analyses should be interpreted cautiously, taking into account the possibility of duplicated studies.

## Introduction

Over the past few decades, there has been an explosive increase in biomedical publications, and the practice of duplicate publication is sometimes problematic in the scientific medical community. According to the International Committee of Medical Journal Editors, duplicate (or redundant) publication can be defined as “the publication of an article that overlaps substantially with one already published in print or electronic media” (2013). Duplicate publication is unethical because it is wasteful of the time, effort, and resources of journals, editors, peer reviewers, readers, libraries, and electronic databases; it also delays the publication time for papers from other researchers (Leopold 2013). This practice calls into question the integrity of science and even distorts the academic reward system. Furthermore, multiple publications of the same work can artificially exaggerate the significance of a particular set of findings or ideas, compromising efforts to conduct effective systematic reviews and meta-analyses.

The incidence of duplicate publications has been measured, and varied incidences of duplicate publication have been demonstrated in previous studies. A total of 7.6% of publications in the orthopedic literature were found to have some degree of redundancy (Gwilym et al. 2004), 1.8–8.5% of articles in the otolaryngology literature (Rosenthal et al. 2003; Bailey 2002), 14% in surgical journals (Schein and Paladugu 2001), < 1% of the plastic surgery literature (Durani 2006), 8.3% of anesthesia and analgesia journals (von Elm et al. 2004), 1.39% of ophthalmology publications, and 2% of hand surgery publications have been identified as duplicate articles (Mojon-Azzi et al. 2004; Chennagiri et al. 2004). In Korea, Kim et al. (Kim et al. 2008) reported that 27 (5.93%) of 455 articles were duplicated.

Meta-analysis is now a commonly used technique for summarizing published data. With the increasing use of meta-analysis, duplicate publication of original research is particularly problematic. Because duplicate publication can result in an inappropriate weighting of the study results, it may result in multiple publication bias (Huston and Moher 1996; Wood 2007; Murphy and Wyllie 2009; Johnson 2006; Tramer et al. 1997). The data from the same patient will be analyzed more than once, leading to biased estimates of treatment efficacy, exaggerated accuracy, and a false impression of drug safety. Thus, both researchers and readers must be alert to the possibility of data overlap due to duplicate publication.

To date, there has been no study of multiple publication bias in meta-analyses. Therefore, the purpose of our study was to assess the incidence and characteristics of duplicate publications in Korea, and to estimate the impact of duplicate publication on meta-analyses.

## Methods

### Systematic search and study selection

The meta-analysis literature written by Korean authors was searched using the online search engines PubMed, KMbase, and KoreaMed. PubMed (http://www.ncbi.nlm.nih.gov) was screened using the key word “Korea” and limited with “meta-analysis” in advanced search for “type of article”. To find meta-analyses not indexed in PubMed, the KMbase (http://kmbase.medric.or.kr) and KoreaMed (http://www.koreamed.org) were searched using “meta-analysis” as a search term. The entire search was performed by 1 librarian, and analysis of selected meta-analyses and included studies was performed independently by 4 investigators.

All investigators screened the titles and abstracts of articles included in the 98 meta-analyses. If potential redundancies, such as the same authors, sample, methodology, results, or conclusions, were identified after screening titles and abstracts, the full article of the suspected duplicate was analyzed, and the contents, methods, subjects, and results were compared separately by 4 reviewers. If any discrepancy in duplication resulted, a consensus was reached by discussion.

### Criteria for a duplicate publication and duplication patterns

In this study, the criteria for duplicate publication of the Editorial Policy Committee of the Council of Science Editors (Editorial Policy Committee of the Council of Science Editors 1996) and a joint statement on duplicate publication established by editors of cardiothoracic journals (Cho et al. 2000) were used to define a duplicate publication. According to these criteria, duplicate publications have a similar hypothesis, similar numbers or sample sizes, identical or nearly identical methodology, similar results, authors in common, and no or little new information (Cho et al. 2000). We regarded the oldest or the largest article of a cluster as the main article. A cross-reference was considered clear if the corresponding article was acknowledged and referenced.

Duplication patterns were described by the classification of von Elm et al. (2004). They suggested the following 4 combinations of duplication patterns: identical samples and identical outcomes (copy), identical samples and different outcomes (fragmentation, salami slicing), increased samples and identical outcomes (imalas), and decreased samples and identical outcomes (disaggregation). To estimate the multiple publication bias, we performed a meta-analysis with and without duplicated data.

## Results

### Identification of duplicates

**Figure 1:**
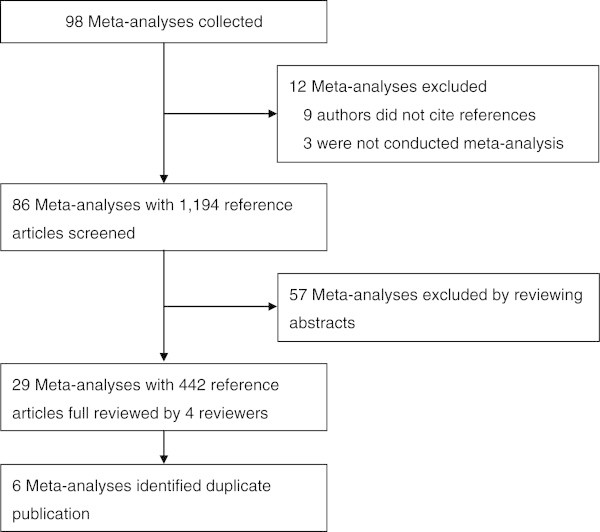
**Flowchart of the screening process for identifying duplicate publications.**

A total of 98 meta-analyses were evaluated, of which 12 were excluded; 9 meta-analyses did not represent reference articles and 3 articles were not meta-analyses. In total, we screened the titles and abstracts of 1194 reference articles from 86 meta-analyses, and excluded 57 meta-analyses and their 752 citations which did not have any duplicate publication, such as the same authors, sample, methodology, results, or conclusions. Finally, 29 meta-analyses were suspected to have duplicate publications. After a thorough full text review of the 442 reference articles of the 29 meta-analyses, 6 articles used in the meta-analyses were identified as duplicates (Figure 1). Based on these results, we estimated that 6 (6.9%) of the 86 analyzed meta-analyses included duplicate publications, and 6 of the 1,194 articles (0.5%) used in the meta-analyses were duplicate publications.

### Characteristics of duplicates and of main articles

**Figure 2:**
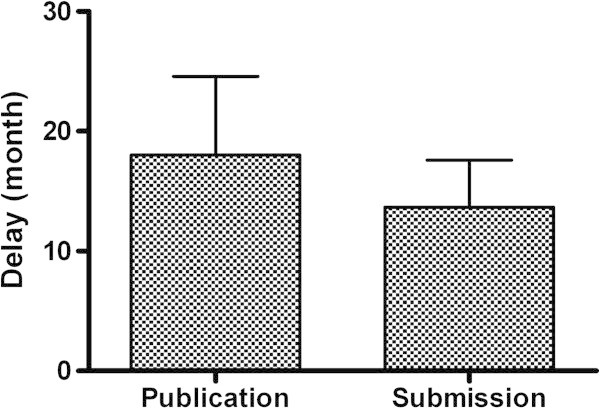
**Delays (in Months) between Publication and Submission of Main Reports and Duplicates.** The median delay in submission between duplicates and main articles was 13 months (range, 6–19 months). The median delay in publication between duplicates and main articles was 18 months (range, 5–48 months).

Table 1 summarizes the baseline characteristics of duplicates and of main articles. Of 6 duplicated articles, 1 was considered a copy (16.6%); 1, a fragmentation (16.6%); 2, imalas (33.3%); and 2, disaggregations of expanded articles written when new data were added to a preliminary article (33.3%). No clear cross referencing was present in the majority of duplicated articles (83.3%). The corresponding author was usually the same, but the other co-authors changed frequently. The number of authors in duplicated articles was higher (mean, 5.3; range, 2–8) than that in main articles (mean, 3.8; range, 1–7). The duplicated articles were published within 2 years of each other, and without subsequent reference to the original publication. The median delay in submission between duplicates and main articles was 13 months (range, 6–19 months). The median delay in publication between duplicates and main articles was 18 months (range, 5–48 months) (Figure 2).

**Table 1:** **Pattern of duplicate publications**

Meta-analysis	Report	Common authorship	No. of subjects	Patterns of duplicate publication	Year of publication	Source of funding	Cross reference
Meta-analysis 1							
	Main	Partial	45		1998	None declared	no
	Duplicate	Partial	45	Identical study sample and reported partially identical outcomes (copy)	2002	None declared	no
Meta-analysis 2							
	Main	Complete	175		1992	None declared	no
	Duplicate	Complete	50	Documented parts of a large trial and reported identical outcomes (disaggregation)	1993	None declared	no
Meta-analysis 3							
	Main	none	100 (case 43)		1996	None declared	no
	Duplicate	none	202 (case 69)	New data were added to a main article and reported identical outcomes (Imalas)	1999	None declared	no
Meta-analysis 4							
	Main	Complete	296		2003	None declared	no
	Duplicate	Complete	197	Duplicates originated from 1 study sample but reported on different outcomes (fragmentation)	2003	None declared	no
Meta-analysis 5							
	Main	Partial	64 (case 11)		1996	None declared	no
	Duplicate	Partial	99 (case 26)	New data were added to a main article and reported almost identical outcomes (Imalas)	1998	None declared	no
Meta-analysis 6							
	Main	Partial	394		2003	None declared	no
	Duplicate	Partial	85	Documented parts of a large trial and reported identical outcomes(disaggregation)	2005	None declared	no

### Impact of duplication

Among the 6 meta-analyses including duplicate publications, we were unable to collect a full dataset of 4 meta-analyses. To evaluate the impact of duplicate publication on the results of meta-analyses, we thus reviewed 2 cases of meta-analyses including duplicate publications (meta-analyses 2 and 3 in Table 1). When a meta-analysis was performed without duplicated data, the mean effect size was 2.0054 (95% confidence interval [CI]: 1.8553, 2.1554) in meta-analysis 3. However, the mean effect size was increased to 2.1394 (95% CI: 1.6248, 2.6570) with duplicated data. The fail-safe number was also increased to 209.1 with duplicated data compared to 203.6 without duplicated data. In the case of meta-analysis 2, there was no difference in the mean effect size without duplicated data compared to the original article. However, the fail-safe number was increased to 17.5 with duplicated data compared to 14.7 without duplicated data.

## Discussion

To the best of our knowledge, the incidence and characteristics of duplicate publications in meta-analyses have not been evaluated at a nationwide level. Our study suggests that 6.9% of the meta-analysis articles that were written by Korean authors included duplicate publications. However, we found only 6 instances of duplicate publication after analyzing 1,194 articles used in meta-analyses that were written by Korean authors. The proportion of multiple publications among the 1,194 collected articles, 0.5%, may be an underestimate, since most duplicated studies were excluded by authors during the process of conducting a meta-analysis. Furthermore, in this study, we did not include meta-analyses not indexed in online databases.

Varied incidences of duplicate publication have been demonstrated in previous studies. In a larger study of the prevalence of redundant publication in the Association Archives of Otolaryngology-Head and Neck Surgery over 8 years, 1,965 authors of 24,353 articles were screened for duplicate publications (Bailey 2002). They found that 201 authors had published 443 redundant articles (1.8% duplication rate). Rosenthal et al. (2003) found that of 492 otolaryngology articles, 42 (8.5%) represented some form of duplication. Gwilym et al. (2004) reported that of 343 “original” articles published in the Journal of Bone and Joint Surgery in 1999, 26 (7.6%) had some degree of redundancy. Gøtzsche (1989) reported 44 (18%) multiple publications among 244 collected studies involving comparisons of nonsteroid anti-inflammatory drugs; the majority of these articles were published within 1 year of each other, and without subsequent reference to the original publication. More recently, Cheung et al. (2013) reported this relatively high incidence of duplicate publication has not significantly changed over 10 years. On the other hand, other studies have reported much lower incidences of duplicate publication. Durani found that 4 (<1%) of 431 original plastic surgery articles had some degree of redundancy (Durani 2006). Chennagiri et al. (2004) found only 14 duplicated articles (2%) among 600 articles published in the Hand Surgery literature. Arrive et al. (2008) reported only 2 instances of redundant publication among 362 original research articles published in Radiology.

In this study, duplicate publications were usually due to disaggregation and overlapping (imalas) publications. An imalas publication is an expansion of a main article through the addition of more data to produce a high-impact article. Disaggregation is the publication of several fragmented parts of a multicenter trial. Reporting the same trial more than once is unacceptable, except in the case of large, multicentre trials with numerous endpoints and following the acceptance of the editor. Generally, the title was modified, and the authors, study sample, and method underwent a minor change in the duplicate publications. All of the duplicate articles in our study were published within 2 years of the main article, and no clear cross referencing was present in the majority (83.3%). However, in 1 case in our study (meta-analysis 3), there was no common authorship. In this case, the study sample, method, outcome, and the department of the authors of the 2 articles were almost identical; we therefore concluded that the articles were overlapping (imalas) publications. The use of different authors for an identical study may be referred as plagiarism, and obscures duplication. Indeed, it may be extremely difficult for reviewers to determine whether 2 papers represent duplicate publications of 1 trial or 2 separate trials when 2 articles reporting the same trial do not share a single common author.

Meta-analyses in the nursing literature had the highest proportion of duplicate publications (33%), and all of the duplicate publications in this study were published before 2005. These cases of duplication might have happened accidentally, or through negligence, rather than by intention. In Korea, the concept of duplicate publications and publication ethics has been raised since 2005 following Professor Hwang’s case (Saunders and Savulescu 2008). Before 2005, Korean researchers might have had little knowledge of publication ethics, which might explain the relatively high rate of duplicate publications.

Detection and exclusion of duplicate data is an important step in conducting a meta-analysis. Our study found an increase in the mean effect size and fail-safe number with duplicated data. This finding means that the effect and reliability was overestimated in a meta-analysis. Multiple publication bias may be an important threat to the validity of meta-analyses. Most importantly, studies with significant results are more likely to lead to multiple publications and presentations (Easterbrook et al. 1991), which makes it more likely that they will be located and included in a meta-analysis. For instance, Tramer et al. (1997) concluded that the inclusion of redundant data in a meta-analysis led to a 23% overestimation of the treatment effectiveness of an antiemetic agent. Murphy and Wyllie (2009) reported that removing the overlapping case would decrease the sample size by 20% in a meta-analysis that quantified the association between initial inappropriate antimicrobial therapy and increased mortality in patients with ventilator-associated pneumonia.

The reasons for duplicate publication may be varied. The authors may have no concept of duplicate publication, or may desire to approach different audience groups. However, in most cases, duplicate publication occurs to boost the author’s career advancement; in other words, the author may feel compelled to publish more for scientific achievement, which is measured by the number of published articles. To prevent duplicate publication, regular education on publication ethics and notifications to members are needed.

There were some limitations in this study. The first limitation is that the judgments and categorizations were subjective in nature. Secondly, the analysis on the effect of duplicate publication on meta-analyses was performed on only 2 meta-analyses including duplicate publications. Furthermore, we did not take into account the confirmation of duplication by the original authors. However, to avoid subjective judgments, 4 reviewers independently reviewed the articles, and if necessary, consensus was reached by discussion. We believe that our findings are helpful in that they will increase the awareness of the problem of duplicate articles and reduce the incidence of duplicate publications in future.

## Conclusion

Our study found only 6 instances of duplicate publication after analyzing 1,194 articles used in meta-analyses written by Korean authors. However, 6.9% of the meta-analyses included duplicate publications. This kind of multiple publication bias will result in an accentuated positive effect of interventions. Our findings suggest that meta-analyses should be interpreted cautiously, taking into account the possibility of duplicated studies.
